# Dichorionic Diamniotic Twin Reduction Surviving the Odds: A Case Report

**DOI:** 10.7759/cureus.79842

**Published:** 2025-02-28

**Authors:** Julia R Legiec, Lauren Lim, Graham Eyeington, Hussain Rawiji

**Affiliations:** 1 Obstetrics and Gynecology, Lake Erie College of Osteopathic Medicine, Bradenton, USA; 2 Pediatrics and Obstetrics, AdventHealth Central Florida, Orange City, USA

**Keywords:** diamniotic twins, dichorionic twins, fetal demise, pregnancy, reduction of pregnancy

## Abstract

A 19-year-old woman presented with reduction of twin pregnancy to singleton pregnancy when premature rupture of the membrane of one dichorionic diamniotic twin at eight weeks of gestation led to premature delivery and fetal demise at 19 weeks of gestation. Despite reactive stress tests performed 11 weeks after rupture of membranes, baby A was delivered via spontaneous vaginal delivery and demised without the assistance of uterotonic medications. Although professional options and limited studies suggested a poor outcome for the remaining fetus, extraordinary measures performed led to a successful, full-term singleton pregnancy.

## Introduction

Twin pregnancies occur in approximately 3% of live births, with dizygotic (fraternal) twins being more common than monozygotic (identical) twins. Determination of amnionicity and chorionicity of twins is based on ultrasonography during the first trimester, preferably after the first seven weeks. The identification of an intertwin membrane with a thickened triangular projection of tissue, about 2 mm, that extends between intertwin membrane layers from a fused dichorionic placenta, best seen at 10-14 weeks gestation is indicative of dichorionic/diamniotic pregnancy. One study stated that the use of lambda sign alone had a sensitivity of 99% and specificity of 95% for predicting dichorionicity [1]. Although dizygotic twins tend to be less stable than monozygotic twins, they face a lower risk of complications than monozygotic twins and higher risk of various complications associated with singleton pregnancies, including the serious concern of preterm birth. The majority of dizygotic twins are dichorionic diamniotic, meaning each twin has its own placenta, blood supply, and amniotic sac due to ovulation and fertilization of two separate oocytes. Compared to monozygotic twins, these twins are less likely to experience complications such as unequal placental sharing, which reduces the risks of twin-twin transfusion syndrome, selective fetal growth restriction, and cord entanglement. Dizygotic twins are also at higher risk of preterm birth rate and death [2]. 

Unfortunately, because each twin has its own set of organs for development, they face a risk of complications that are roughly double that of singleton pregnancies. Additionally, since the twins share a single uterus, any issue affecting one twin, such as premature demise, preeclampsia, or chorioamnionitis, can have significant implications for the other twin. In such cases, the remaining fetus is at increased risk and requires close monitoring. Timely intervention and delivery may be necessary to prevent fetal loss or maternal morbidity. If this occurs before the fetus has reached viable gestational age to survive ex-utero, both fetuses may be lost and lead to premature demise of the entire pregnancy and high risk of postpartum depression [3]. In situations where the surviving fetus cannot survive prompt delivery, if possible, the pregnancy should continue to be monitored closely and plan on induction of labor at 36 weeks.

One multiple case study investigating outcomes and management of fetal death in twin pregnancy found that in dichorionic twins, the prognosis for the surviving twin is good compared to monochorionic monoamniotic twins. They have discovered that the surviving fetal had a poor prognosis due to damage to placental vascular anastomosis, resulting in permanent neurological damage [4]. This study also states that the main risk factor for fetal demise in one twin of dizygotic pregnancy was fetal immaturity of the surviving twin. Another more recent retrospective cohort study that was conducted supported that fetal reduction from twins to singleton may be beneficial for the remaining fetus [5]. This study found that twin to singleton reduction by transabdominal intracardiac injection of KCl using ultrasound guidance leads to decreased prematurity of surviving twin, prolongs gestation by one week, and overall has more favorable outcomes than ongoing, non-reduced twins. 

This case report presents a unique scenario in which one dichorionic diamniotic twin is delivered prematurely due to premature rupture of membranes at eight weeks, resulting in the spontaneous vaginal delivery of twin A at 16 weeks of gestation. The decision to leave the placenta in utero and administer empiric antibiotics may play a crucial role in improving the outcome for the surviving twin, potentially offering an answer to a successful full-term singleton pregnancy.

## Case presentation

**Figure 1:**
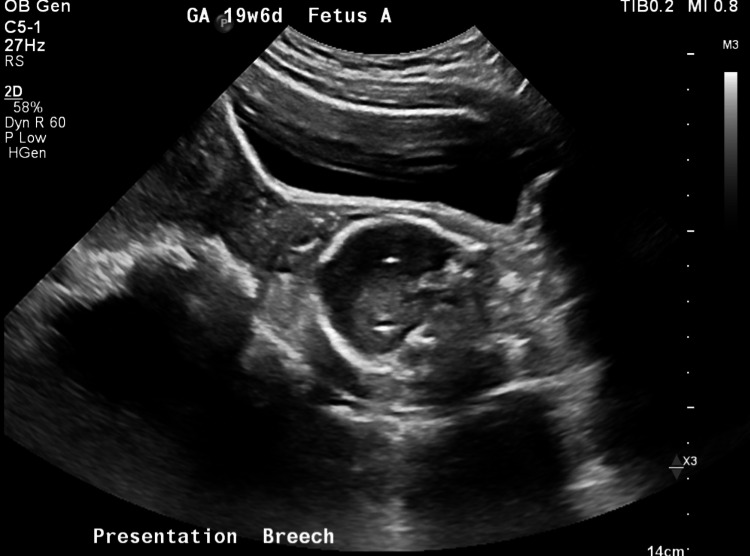
Transabdominal US image depicting baby A in the patient’s dilated cervix at 19 weeks and 6 days gestation.

**Figure 2:**
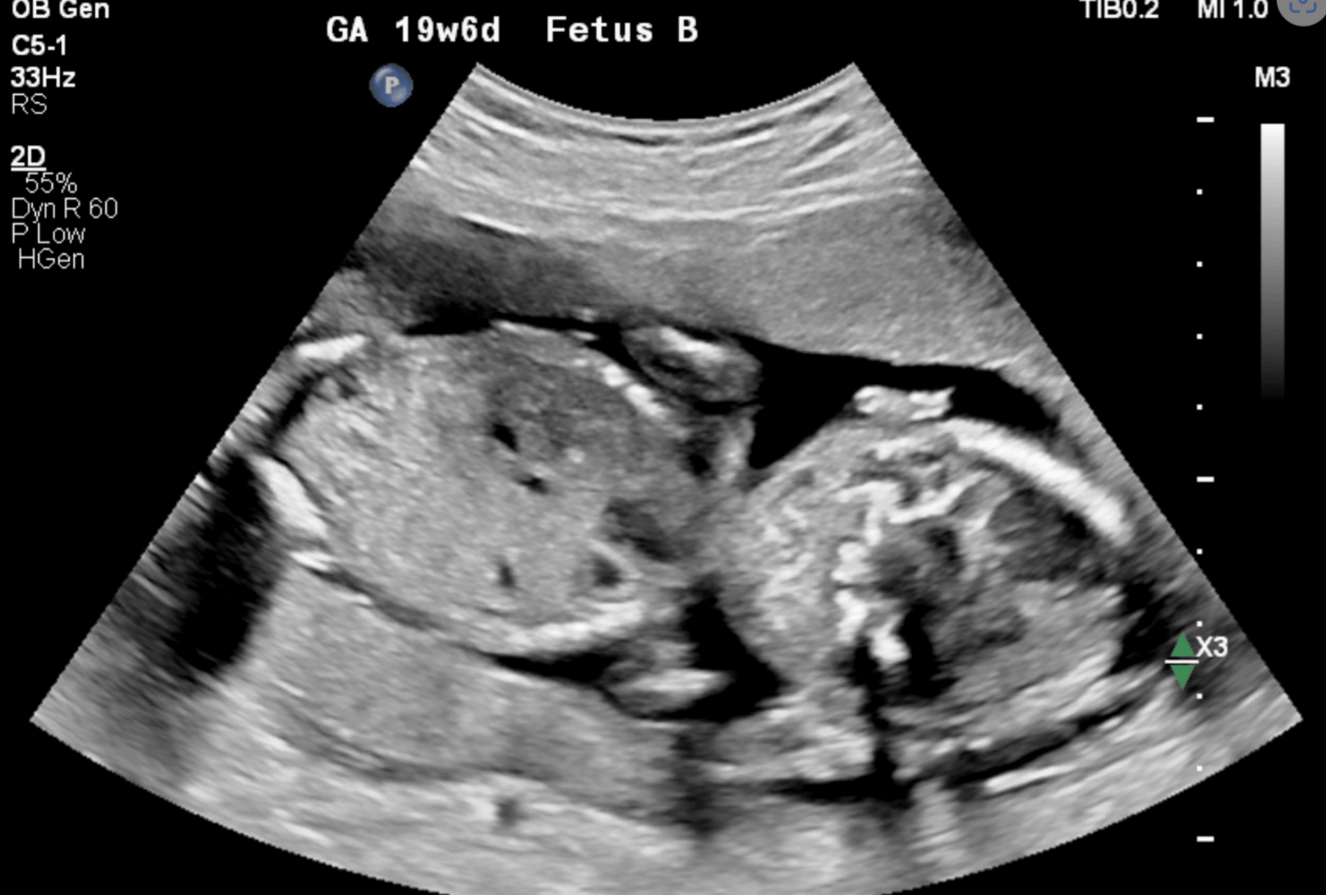
Transabdominal US image showing baby B in utero at 19 weeks and 6 days gestation.

**Figure 3:**
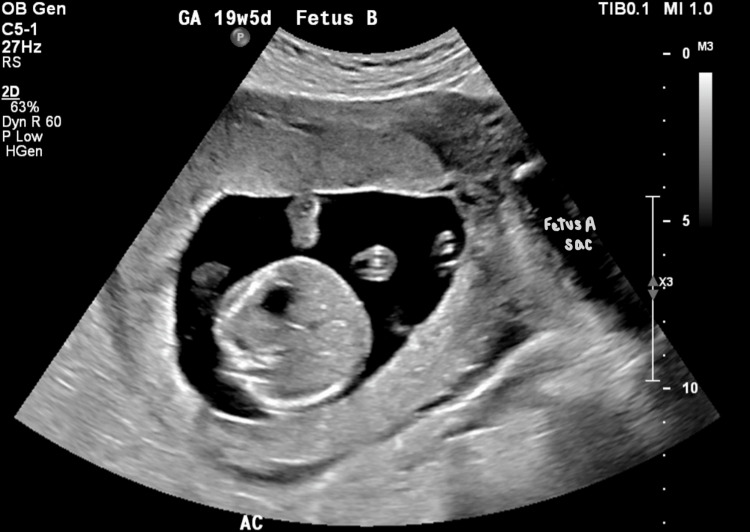
Transabdominal US image depicting baby B in its amniotic sac and baby A’s amniotic sac to the left.

The patient was a 19-year-old primigravida woman who presented to the AdventHealth FISH Memorial emergency department with premature rupture of membranes with dichromatic diamniotic 19-week four-day pregnancy. Baby A was a girl with anhydramnios since 15 weeks gestation secondary to premature rupture of membranes and baby B was a boy. The patient had a positive history of vaginal bleeding since eight weeks of pregnancy with diagnosis of subchorionic hematoma. During the physical exam, there was blood present in the cervix with understandable discomfort from the patient. Complete blood count showed low red blood cell count, low hemoglobin, and low hematocrit indicating anemia. Transabdominal ultrasonography was performed, showing baby A was situated in the lower dilated cervix in the breech position with anhydramnios and a small femoral length, and baby B was in the breech position with normal AFI (Figures 1-3). The patient was made aware of the poor survival chances of baby A and that within the next 48 hours, there was a high chance that baby A would most likely be aborted via spontaneous vaginal delivery. The patient was also aware that the chances for baby B’s survival were minimal; however, we were going to do everything in our abilities to attempt to keep baby B in utero. For further clarity on the situation, the hospital on call family medicine hospitalist was contacted. They had a detailed discussion with the OBGYN that the likelihood of baby B reaching a viable gestational age outside the womb was less than 10%, with a prognosis of a 90% chance of poor outcome.

After 13 hours of labor, baby A was partially expelled from the vagina. Epidural was requested to aid in the delivery of baby A. Once the epidural was placed, baby A was delivered deceased with a bruised arm that had been partially out of the vagina. The patient and family had time to mourn with the baby A. After delivery, the placental cord was cut high in the vagina and was sutured with 3-0 vicryl to decrease the chance of chorioamnionitis, prevent the placenta from being expelled, and ensure that there would be minimal uterine contraction to prevent the delivery of baby B. Betadine was placed in and around the vagina immediately after delivery, and the patient was started on broad-spectrum antibiotics including zithromax and indomethacin.

The patient was offered to return home immediately after delivery and to return if she felt she was in labor; however, the patient wanted to stay to make sure she had the best chances of survival for baby B. The patient stayed postpartum for one week, receiving azithromycin (500mg) q24f for the first five days, Piperacillin-Tazo 4.5 g IV q8h SCH and estrogen tablets, vaginally, daily. Every other day the umbilical cord was examined and shortened to reduce exposure to bacteria. When the patient felt ready to leave, she was discharged with progesterone 200 mg vaginally daily as well as continuation of oral antibiotics.

After almost three weeks after discharge, hospital records show that the patient experienced a large expulsion of vaginal discharge and blood, suspected to be the complete placental passage of baby A. Recent documentation of this pregnancy revealed that a little under three months after discharge, the patient was experiencing premature rupture membranes of baby B, described as a gushing of the fluid with positive scant fluid on pelvic examination. Once the patient was examined at AdventHealth FISH Memorial, the patient was transferred to a high-risk pregnancy care facility, Orlando Health Winnie Palmer Hospital for Women and Babies. One month after this episode, the patient was scheduled for a cesarean section due to baby B presenting in the breech position. The baby was born without complications at 35 weeks gestation.

## Discussion

Clinically navigating the treatment of a remaining fetus in utero after the demise of the other twin may pose a challenging situation to navigate as a clinician, as well as a patient. In situations where it may be possible to salvage the life of the remaining fetus, it is imperative that the clinician and patient decide on a plan in a timely manner in order to prevent fetal loss and reduce maternal morbidity. The effect on the patient’s health, emotional, and physical well-being should be considered when making clinical decisions, as the loss of one fetus as well as potentially losing a second can cause significant distress and an increased risk of developing postpartum depression. In this case, the patient was able to vaginally deliver baby B at 35 weeks despite the fetal demise of baby A at 19 weeks. Despite the unfortunate event of the passing of baby A, this patient’s journey of transitioning from dichorionic, diamniotic twins to a singleton pregnancy is noteworthy in that it may serve as a clinical guide for physicians and their decision-making when presented with unconventional patient populations similar to this one. 

The patient’s ability to deliver the second fetus without complications was considered to be a very rare occurrence. Premature rupture of membranes is a significant risk factor for premature labor, chorioamnionitis, and oligohydramnios. If one of the twins undergoes premature rupture of membranes, the surviving twin has a lower chance of survival due to the risks mentioned above. In addition, although dichorionic, diamniotic twins contain their own placentas and amniotic sacs, the maternal response to fetal demise of one twin may trigger an inflammatory response that can be harmful to the remaining fetus [4]. In general, there is an increased risk of preterm labor in twin pregnancies in comparison to singleton pregnancies. It is important to note that between monochorionic and dichorionic twins, monochorionic twins statistically have an increased risk for preterm labor due to risk of twin-twin transfusion syndrome and unequal placental sharing, which is not a concern in dichorionic twins since they contain their own placentas [2]. In 2019, 6,225 twin pregnancies were analyzed and found that in dichorionic twins, approximately 2.3% of pregnancies experienced the loss of one or both fetuses prior to 24 weeks when both were alive at 11 to 13 weeks. There was a higher rate of loss prior to 24 weeks in monochorionic, diamniotic twins at 7.4% [2]. The study emphasized the potential importance of using endolaser therapy specifically in monochorionic diamniotic twins, which would photocoagulate abnormal vessels within the shared placenta and stop the unequal blood sharing between the two twins in order to increase the survival of both [2].

It is important to acknowledge the fact that although this patient’s risk of preterm labor was higher compared to singleton pregnancies, the chances of survival in this dichorionic, diamniotic pregnancy were still higher compared to if she had monochorionic twins instead. Although twin-twin transfusion syndrome was not a concern in this patient, infection and preterm labor were still a possible implication in this pregnancy and this was avoided as best as possible with the use of antibiotics and progesterone. Despite treatment being seemed limited for this patient, it is important to acknowledge that the simple measures taken may have led to the patient’s successful delivery of the remaining fetus.

There have been studies done exploring the potential benefits and risks of intentionally reducing twin pregnancies to a singleton pregnancy in order to prevent adverse outcomes, such as the one initially mentioned in the introduction [5]. Currently, there is reasonable evidence to believe that reduction to singleton pregnancy may allow for reduced risk of preterm birth and lead to greater birthweights and greater likelihood in prolonging the pregnancy period of the remaining fetus when compared to non-reduced twins. However, the intentional fetal reduction of twins to singleton pregnancy has been a controversial topic for quite some time, due to contradicting evidence that it may also lead to greater chances of preterm deliveries and fetal demise [5]. In this study, 98 reduced pregnancies were compared to the control group consisting of 222 ongoing twins [5]. For the experimental group, there were different indications for the procedure. These included anatomic malformations, aneuploidies, increased risk for preterm delivery, and lastly due to the patient’s personal request. In terms of the procedure of fetal reduction, potassium chloride injection was administered transabdominally with a 20 Gauge needle into the one of the fetuses' heart until asystole was obtained, using a real time ultrasound for needle guidance for accuracy. At the end of the study, it was concluded that fetal reduction from twins to a singleton pregnancy has the potential to reduce preterm pregnancies before 37 weeks [5]. However, this may not be the outcome in more severe perinatal complications and is still very controversial.

Further studies have explored the prognosis of the remaining fetus in a twin pregnancy after the demise of one fetus according to its location during the first trimester of pregnancy [6]. In this study, two groups were separated based on the location of the demised fetus. Group 1 included twin pregnancies in which the demised fetus was considered to be the presenting fetus, meaning that it was the closest to the birth canal. Group 2 included twin pregnancies in which the demised fetus was located further from the birth canal in relation to the remaining fetus. The study found that the gestational age of the remaining fetuses from Group 1 were noticeably earlier than those in Group 2, meaning that in pregnancies in which the demised fetus is closest to the birth canal, the mother is more likely to undergo labor earlier than usual. The average delivery time for Group 1 was 33.8 weeks and 37.3 weeks for group 2. Group 1 also had higher rates of preterm births at 22% compared to 4.5% in Group 2 [6]. It was suspected that when the demised fetus is also the presenting fetus, there is a high chance of them triggering an inflammatory response or infection near the cervix, leading to a higher chance of premature labor. In addition, the body’s natural response to a demised fetus may induce contractions that can cause premature labor. Another study found that delivery occurred substantially earlier in pregnancies in which the demised fetus was the presenting fetus. In addition, there was an increased rate of anomalies found in the surviving twin when the presenting twin was the demised twin. It was hypothesized that a disintegrating fetus closer to the cervical canal may be an inciting source for chorioamnionitis and premature rupture of membranes, as well as reduce the integrity of the cervical tissue, potentially inducing premature labor [7]. It has also been theorized that the remaining fetus may have a higher risk of experiencing intrauterine growth restriction and pre-eclampsia [8]. In the case of our patient, baby A was both the presenting and demised fetus. Baby B was the remaining fetus and was in breech position. There was concern that the passage of the demised fetus and placenta would induce uterine contractions and expulsion of the remaining fetus. Because of this, the placenta of the demised fetus was kept inside of the uterus as long as possible to prevent premature labor of the remaining fetus.

## Conclusions

This case demonstrates an unusual presentation of fetal demise due to premature rupture of the amniotic membrane of one of the fetuses, in a dichorionic diamniotic pregnancy. It highlights the complexities and the high-risk management that go into optimizing survival outcomes for the remaining fetus while simultaneously prioritizing the mother’s health and well-being. Even though the chances of survival in the remaining fetus were low, meticulous care was taken to preserve the intrauterine environment in order to prolong gestation and improve survival outcomes. These measures included prompt treatment of the patient with antibiotics and progesterone in order to prevent infection and premature labor of the remaining fetus. This case report highlighted the fact that the chances of survival of the remaining fetus after premature rupture of membranes and demise of the first fetus are very low. Especially when the demised fetus is also the presenting fetus, there seems to potentially be an increased risk of infection, premature labor, and anomalies in the surviving fetus. In this case report, however, the patient was able to carry her remaining fetus to term despite the odds being against her. A combination of antibiotics, progesterone, a strong patient-physician relationship, and an optimistic outlook with regard to her journey may have contributed to this patient’s miraculous outcome. Considering there is a scarcity of documented cases similar to this one, we encourage more future research and case reports to explore other treatment options that may be useful in a patient population similar to this one.
